# Deciphering associations between three RNA splicing-related genetic variants and lung cancer risk

**DOI:** 10.1038/s41698-022-00281-9

**Published:** 2022-06-30

**Authors:** Wenjun Yang, Hongliang Liu, Ruoxin Zhang, Jennifer A. Freedman, Younghun Han, Rayjean J. Hung, Yonathan Brhane, John McLaughlin, Paul Brennan, Heike Bickeboeller, Albert Rosenberger, Richard S. Houlston, Neil E. Caporaso, Maria Teresa Landi, Irene Brueske, Angela Risch, David C. Christiani, Christopher I. Amos, Xiaoxin Chen, Steven R. Patierno, Qingyi Wei

**Affiliations:** 1 International Center for Aging and Cancer, Pathology Department of the First Affiliated Hospital, Hainan Medical University, Haikou, 571199 China; 2 Duke Cancer Institute, Duke University Medical Center, Durham, NC 27710 USA; 3 Ningxia Human Stem Cell Research Institute, the General Hospital of Ningxia Medical University, Yinchuan, 750004 China; 4 Department of Population Health Sciences, Duke University School of Medicine, Durham, NC 27710 USA; 5 School of Public Health, Fudan University; Key Laboratory of Public Health Safety, Ministry of Education, Shanghai, 200032 China; 6 Yiwu Research Institute of Fudan University, Yiwu, Zhejiang 322000 China; 7 Department of Medicine, Division of Medical Oncology, Duke University School of Medicine, Durham, NC 27710 USA; 8 Institute for Clinical and Translational Research, Baylor College of Medicine, Houston, TX 77030 USA; 9 Lunenfeld-Tanenbaum Research Institute, Sinai Health System, Toronto, ON Canada; 10 Public Health Ontario, Toronto, ON M5T 3L9 Canada; 11 International Agency for Research on Cancer, World Health Organization, Lyon, 69372 France; 12 Department of Genetic Epidemiology, University Medical Center Göttingen, Göttingen, 37073 Germany; 13 Division of Genetics and Epidemiology, the Institute of Cancer Research, London, SW7 3RP UK; 14 Division of Cancer Epidemiology and Genetics, National Cancer Institute, National Institutes of Health, Bethesda, MD 20892 USA; 15 Helmholtz Centre Munich, German Research Centre for Environmental Health, Institute of Epidemiology, Neuherberg, 85764 Germany; 16 Department of Molecular Biology, University of Salzburg, Salzburg, 5020 Austria; 17 Massachusetts General Hospital, Boston, MA 02114 USA; 18 Department of Environmental Health, Harvard School of Public Health, Boston, MA 02115 USA; 19 Department of Medicine, Baylor College of Medicine, Houston, TX 77030 USA; 20 Cancer Research Program, Julius L. Chambers Biomedical Biotechnology Research Institute, North Carolina Central University, Durham, NC 27707 USA; 21 Duke Global Health Institute, Duke University Medical Center, Durham, NC 27710 USA

**Keywords:** Non-small-cell lung cancer, Cancer epidemiology

## Abstract

Limited efforts have been made in assessing the effect of genome-wide profiling of RNA splicing-related variation on lung cancer risk. In the present study, we first identified RNA splicing-related genetic variants linked to lung cancer in a genome-wide profiling analysis and then conducted a two-stage (discovery and replication) association study in populations of European ancestry. Discovery and validation were conducted sequentially with a total of 29,266 cases and 56,450 controls from both the Transdisciplinary Research in Cancer of the Lung and the International Lung Cancer Consortium as well as the OncoArray database. For those variants identified as significant in the two datasets, we further performed stratified analyses by smoking status and histological type and investigated their effects on gene expression and potential regulatory mechanisms. We identified three genetic variants significantly associated with lung cancer risk: rs329118 in *JADE2* (*P* = 8.80E−09), rs2285521 in *GGA2* (*P* = 4.43E−08), and rs198459 in *MYRF* (*P* = 1.60E−06). The combined effects of all three SNPs were more evident in lung squamous cell carcinomas (*P* = 1.81E−08, *P* = 6.21E−08, and *P* = 7.93E−04, respectively) than in lung adenocarcinomas and in ever smokers (*P* = 9.80E−05, *P* = 2.70E−04, and *P* = 2.90E−05, respectively) than in never smokers. Gene expression quantitative trait analysis suggested a role for the SNPs in regulating transcriptional expression of the corresponding target genes. In conclusion, we report that three RNA splicing-related genetic variants contribute to lung cancer susceptibility in European populations. However, additional validation is needed, and specific splicing mechanisms of the target genes underlying the observed associations also warrants further exploration.

## Introduction

RNA splicing is a process in which an mRNA precursor can produce multiple mRNA isoforms that dramatically diversify the transcriptome and the proteome in eukaryotic cells^1–3^. In humans, it is estimated that up to 94% of the genes are differentially spliced, and the resulting protein isoforms can contribute to proteome complexity and have diverse or even opposite biological functions, with profound consequences on cellular processes and phenotypes^4^. Several studies have described aberrant or dysregulated RNA splicing events as well as alternative RNA splicing events, which can frequently be affected by genetic variants and mutations, that contribute to cancer cell phenotypes by directly or indirectly regulating the genome, epigenome, transcriptome, and proteome^5–9^. Aberrant RNA splicing has been implicated in the ancestry-related biology of cancer disparities and as a potential source of novel targets for precision oncology^10^. The role of aberrant splicing as a primary cause of Mendelian diseases has been widely accepted by decades of related studies^11–13^. However, much less has been reported and appreciated regarding the extent of physiological RNA splicing variation among human populations and the phenotypic variability and disease susceptibility affected by them in humans^5^.

Lung cancer is the most common malignancy in humans, leading the causes of cancer death worldwide^14^. Non-small cell lung cancer (NSCLC) is the most prevalent and heterogeneous subtype of lung cancer, including lung adenocarcinoma (LUAD) and lung squamous cell carcinoma (LUSC)^15^. Although a large number of germline mutations within cancer susceptibility genes have been reported, genetic etiology of lung cancer is still not fully understood^16,17^. In addition, until recently, despite the important role of RNA splicing in cancer, limited efforts have been made in the genome-wide profiling of RNA splicing-related variation in cancer patients, including in NSCLC^15^.

We have previously reported single nucleotide polymorphisms (SNPs) in RNA splicing-related regulatory sequences in prostate cancer stemness-associated genes that are associated with race and/or survival^18,19^. To conduct a comprehensive characterization of a genome-wide profile of common lung cancer genetic susceptibility loci associated with RNA splicing, we first performed a meta-analysis to discover potential RNA splicing-related SNPs using summary statistics from eight published lung cancer genome-wide association studies (GWASs) from the Transdisciplinary Research in Cancer of the Lung (TRICL) and the International Lung Cancer Consortium (ILCCO)^20^. Those significant SNPs discovered were then validated using data from the OncoArray platform that provides an unprecedented opportunity for additional de novo discovery as well as for fine mapping of lung cancer susceptibility^16,21^. For those identified SNPs that were found to be significant in both discovery and validation datasets, we further performed stratified analyses by smoking status and histological type and investigated their effects on gene expression and potential regulatory mechanisms in cell lines and tissues by using the available genomic and genetic data from multiple public databases.

## Results

### Meta-analysis of the main effects

**Fig. 1:**
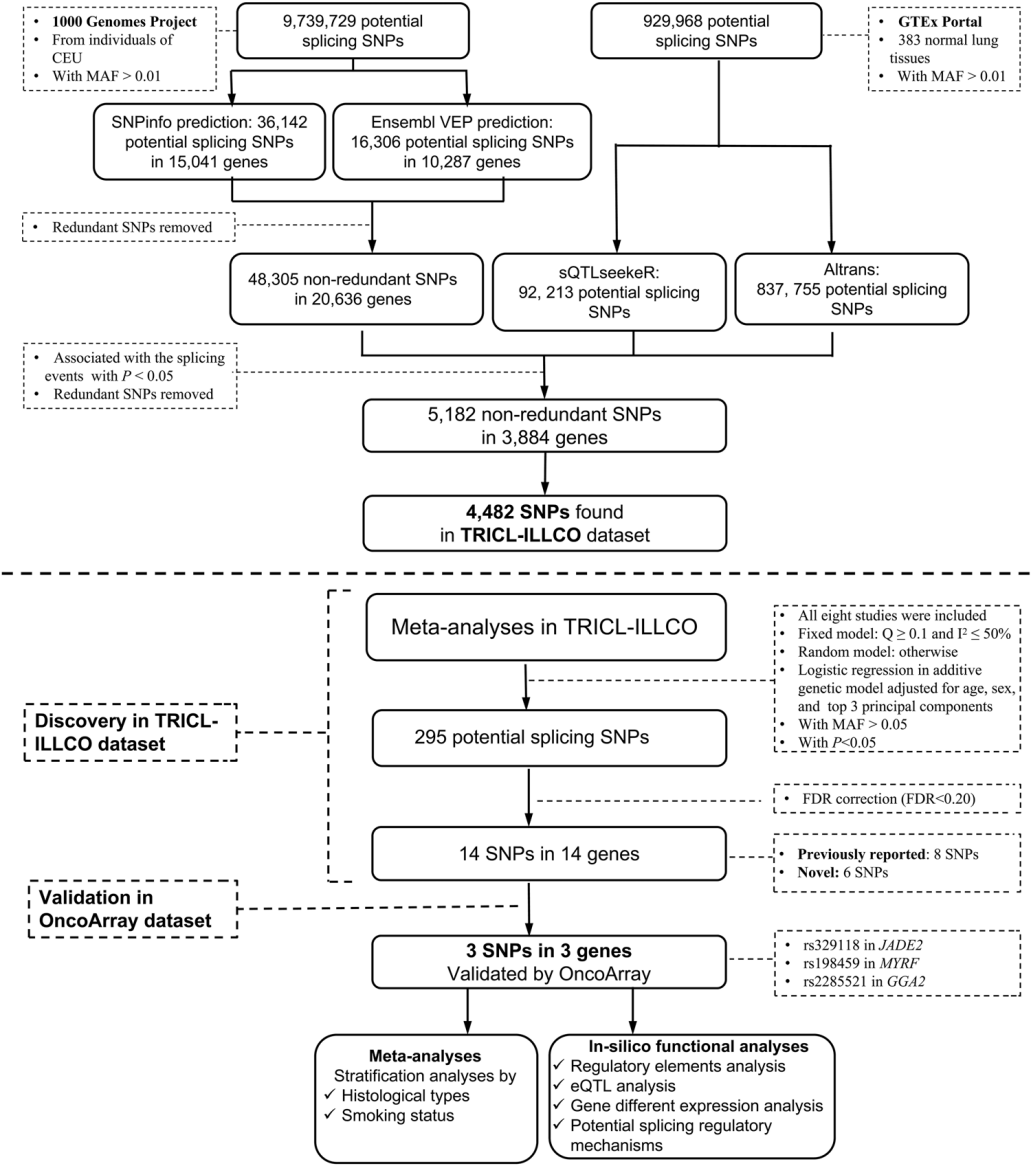
Study flowchart. CEU Caucasian, MAF minor allele frequency, FDR false discovery rate, eQTL expression quantitative trait loci.

We focused on the joint analyses of both TRICL-ILLCO and OncoArray sets, representing the largest sample size of European ancestry to date. The study populations (29,266 lung cancer cases and 56,450 non-cancer controls) for the eight GWASs from TRICL-ILLCO consortia (i.e., ICR, MDACC, IARC, NCI, Toronto, GLC, Harvard, and deCODE GLC) and OncoArray dataset and the demographic characteristics of the final dataset are summarized in Supplementary Table 1, and the work workflow is depicted in Fig. 1.

**Fig. 2:**
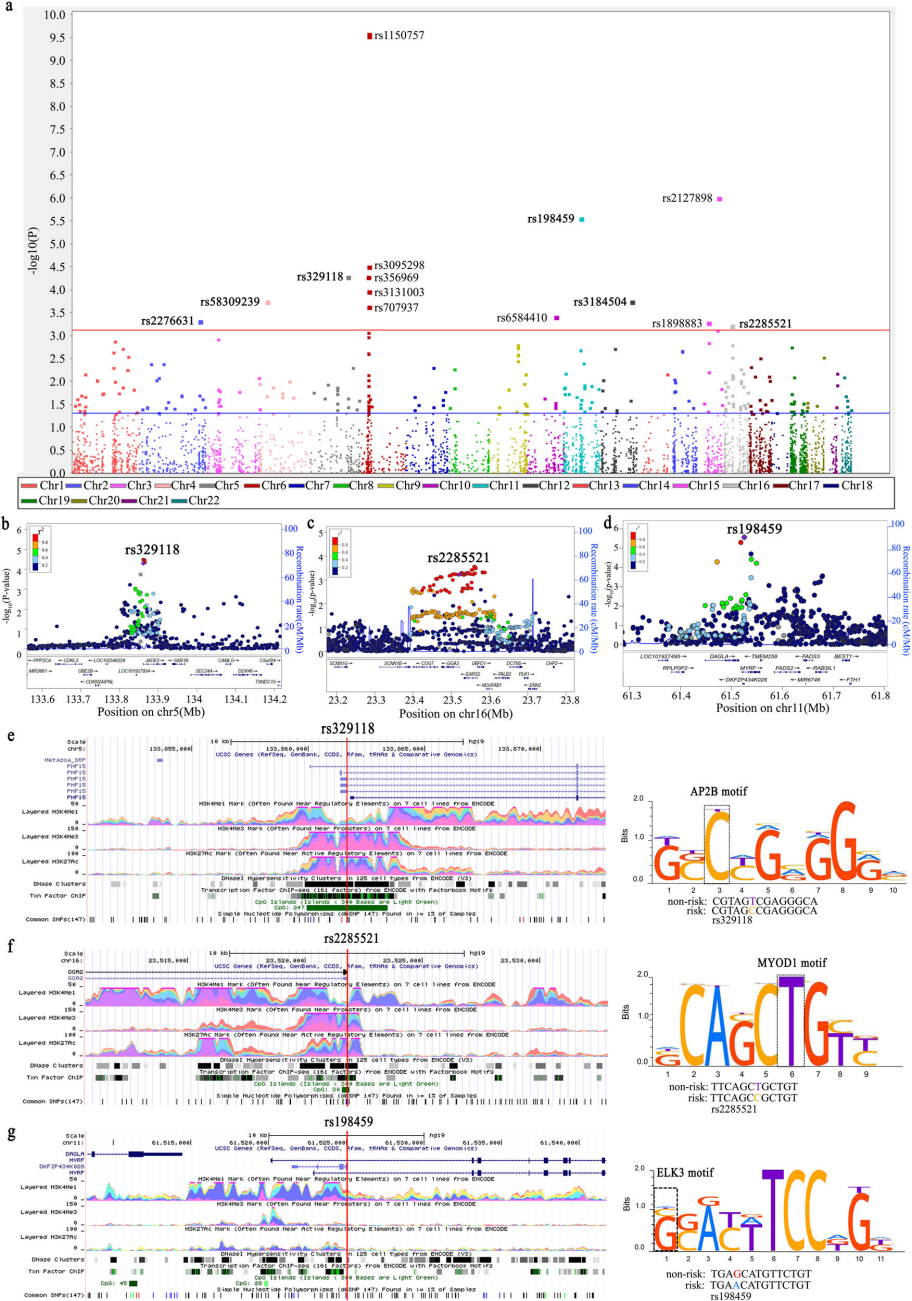
Association results and functional prediction of lung cancer risk-associated potential splicing SNPs. **a** Manhattan plot of the overall results. There were 295 SNPs related to RNA splicing with a nominal *P* < 0.05, 14 of which remained with FDR < 0.20. The *x*-axis indicates the chromosome number and the *y*-axis shows the association *P* values with lung cancer risk (as −log10 *P* values). The horizontal blue line represents *P* values of 0.05, while the red line indicated the FDR threshold 0.20. Regional association plot, which shows the LD between the top SNP rs329118 on *JADE2* (**b**), rs2285521 on *GGA2* (**c**), and rs198459 on *MYRF* (**d**), and other SNPs in the region of 500 kb up- or downstream of the top SNP. Locations, functional prediction, and position weight matrix based Sequence Logo of three SNPs. *JADE2* rs329118 (**e**) and *GGA2* rs2285521 (**f**) are located within one CpG island and presented strong signals of active enhancer and promoter functions (indicated by H3K4 methylation, histone modification H3K27 acetylation, and DNase hypersensitivity, respectively). *MYRF* rs198459 (**g**) is located within one CpG island and presented strong signals of active enhancer and promoter functions (indicated by H3K4 methylation and DNase hypersensitivity, respectively). The panels were adapted from the UCSC Genome Browser. Three SNPs are located on the AP2B motif (**e**), the MYOD1 motif (**f**), and the ELK3 motif (**g**), respectively.

The overview of overall association results is shown in the Manhattan plot (Fig. 2a). We found that there were 295 SNPs related to RNA splicing with a nominal *P* < 0.05, of which 14 SNPs remained with a false discovery rate (FDR) < 0.20 for multiple testing correction. There was no heterogeneity observed for the effect estimates of these 14 SNPs from the eight GWASs (Supplementary Table 2).

Among the 14 SNPs identified, six were unreported, which were then validated using the OncoArray dataset (14,803 cases and 12,262 controls), and three SNPs reached a nominal *P* < 0.05 (Table 1). All three SNPs were imputed with the overall imputation information/ R-squared in the eight TRICL-ILLCO GWAS presented in Supplementary Table 3. After a final combined analysis, as shown in Table 1, *JADE2* rs329118 T>C was found to be associated with a significantly decreased risk of lung cancer [odds ratio (OR) = 0.94, 95% confidence interval (CI) = 0.92–0.96, *P* = 8.80 × 10^−9^], while two other SNPs were associated with a significantly increased risk of lung cancer (*GGA2* rs2285521 C>T: OR = 1.08, 95% CI = 1.05–1.11, *P* = 4.43 × 10^−8^ and *MYRF* rs198459 G>A: OR = 1.07, 95% CI = 1.04–1.11, *P* = 1.60 × 10^−6^). No heterogeneity was observed for the effect estimates of these three SNPs from the eight GWASs and the OncoArray dataset (Supplementary Fig. 1). The regional association plots of these three SNPs are shown in Fig. 2b–d.

**Table 1:** SNPs associated with lung cancer risk discovered in TRICL-ILLCO consortia and validated in OncoArray dataset.

SNP rs#	Chr	Position	Alleles^a^	Encode gene	Discovery in TRICL-ILLCO				Validation in OncoArray			Combination	
					MAF	OR (95% CI)^b^	*P*^*b*^	FDR	MAF	OR (95% CI)^b^	*P*^*b*^	OR (95% CI)^b^	*P*^*b*^
rs329118	5	133861663	T/C	*JADE2*	0.42	0.93 (0.90–0.96)	5.03E−05	0.032	0.43	0.94 (0.91–0.97)	5.18E−04	0.94 (0.92–0.96)	8.80E−09
rs2285521	16	23521780	C/T	*GGA2*	0.16	1.09 (1.04–1.14)	5.90E−04	0.165	0.15	1.07 (1.02–1.13)	4.23E−03	1.08 (1.05–1.11)	4.43E−08
rs198459	11	61525020	A/G	*MYRF*	0.22	1.11 (1.06–1.16)	2.71E−06	0.003	0.22	1.05 (1.01–1.10)	0.018	1.07 (1.04–1.11)	1.60E−06
rs58309239	4	25443366	G/T	*LOC105374536*	0.05	0.85 (0.78–0.93)	1.77E−04	0.079	0.05	0.97 (0.90–1.05)	0.466	0.91 (0.84–0.99)	4.72E−04
rs3184504	13	111884608	T/C	*SH2B3*	0.48	0.93 (0.90–0.97)	1.75E−04	0.079	0.49	0.99 (0.96–1.03)	0.617	0.96 (0.94–0.98)	6.78E−04
rs2276631	2	219249013	T/C	*SLC11A1*	0.26	0.93 (0.90–0.97)	4.72E−04	0.159	0.25	1.00 (0.96–1.04)	0.938	0.96 (0.93–0.98)	8.45E−04

Abbreviations: *SNP*, single nucleotide polymorphism, *Chr* Chromosome, *MAF*, minor allele frequency, *OR* odds ratio, *CI* confidence interval, *FDR* false discovery rate.

^a^Effect allele/Reference allele.

^b^Adjusted for top principle components.

We then performed functional prediction for these three significant SNPs by using three bioinformatics tools (SNPinfo, regulomDB, and HaploReg) to predict their potential effects on gene expression or biological functions for further analysis (Supplementary Table 4).

### Stratified analyses

To assess whether histological types of lung cancer may be impacted by different genetic factors, we performed stratified analyses by LUAD and LUSC. By using 11,273 LUAD and 7,426 LUSC from both the TRICL-ILLCO and OncoArray datasets, we found that the effects of all three SNPs (*JADE2* rs329118, *GGA2* rs2285521, and *MYRF* rs198459) were more evident in LUSC (OR = 0.91, 95% CI = 0.88–0.95; OR = 1.13, 95% CI = 1.08–1.19; OR = 1.08, 95% CI = 1.03–1.13, respectively) than in LUAD (OR = 0.95, 95% CI = 0.91–0.98; OR = 1.04, 95% CI = 0.99–1.09; OR = 1.05, 95% CI = 1.00–1.12, respectively)). However, no significant heterogeneity was found for either of the two histological strata for these three SNPs (Table 2 and Supplementary Fig. 1).

**Table 2:** Associations between three SNPs and lung cancer risk stratified by histologic types and smoking status in all eight lung cancer GWASs and OncoArray dataset.

Study	Case	Control	rs329118		rs2285521		rs198459	
			OR (95% CI)	*P*	OR (95% CI)	*P*	OR (95% CI)	*P*
Overall			*I*^2^ = 0.0%, *P* = 0.988		*I*^2^ = 0.0%, *P* = 0.866		*I*^2^ = 19.7%, *P* = 0.267	
ICR	1952	5200	0.92 (0.86–1.00)	0.038	1.04 (0.94–1.15)	0.480	1.03 (0.94–1.13)	0.532
MDACC	1150	1134	0.95 (0.84–1.07)	0.407	1.17 (0.99–1.39)	0.064	1.18 (1.00–1.40)	0.047
IARC	2533	3791	0.92 (0.85–0.99)	0.027	1.11 (1.01–1.23)	0.039	1.10 (1.00–1.21)	0.054
NCI	5713	5736	0.94 (0.89–0.99)	0.023	1.10 (1.02–1.18)	0.012	1.15 (1.08–1.23)	4.00E−05
Toronto	331	499	0.93 (0.74–1.17)	0.528	0.97 (0.70–1.33)	0.839	1.09 (0.83–1.42)	0.548
GLC	481	478	0.88 (0.73–1.07)	0.193	1.01 (0.78–1.29)	0.969	1.14 (0.89–1.44)	0.295
Harvard	984	970	0.93 (0.82–1.06)	0.298	1.10 (0.91–1.32)	0.350	0.98 (0.84–1.14)	0.799
deCODE	1319	26380	0.94 (0.87–1.02)	0.135	1.01 (0.90–1.14)	0.867	1.02 (0.91–1.14)	0.728
OncoArray	14360	11555	0.94 (0.91–0.97)	5.18E−04	1.07 (1.02–1.13)	4.23E−03	1.05 (1.01–1.10)	0.018
Overall	28823	55743	0.94 (0.92–0.96)	8.80E−09	1.08 (1.05–1.11)	4.43E−08	1.07 (1.04–1.11)	1.60E−06
Adenocarcinoma			*I*^2^ = 0.0%,*P* = 0.783		*I*^2^ = 0.0%, *P* = 0.933		*I*^2^ = 26.5%, *P* = 0.209	
ICR	465	5200	1.01 (0.88–1.15)	0.938	1.07 (0.88–1.29)	0.504	1.03 (0.87–1.22)	0.714
MDACC	619	1134	0.93 (0.80–1.08)	0.328	1.10 (0.90–1.35)	0.341	1.17 (0.96–1.42)	0.130
IARC	517	2824	0.91 (0.79–1.04)	0.163	1.15 (0.96–1.37)	0.125	1.08 (0.90–1.29)	0.425
NCI	1841	5736	0.94 (0.87–1.01)	0.103	1.02 (0.92–1.14)	0.718	1.16 (1.06–1.28)	0.002
Toronto	90	499	0.85 (0.61–1.21)	0.370	1.10 (0.67–1.79)	0.713	0.89 (0.58–1.36)	0.596
GLC	186	478	0.77 (0.59–1.00)	0.047	0.97 (0.69–1.35)	0.842	0.90 (0.65–1.25)	0.528
Harvard	597	970	0.94 (0.81–1.09)	0.391	1.11 (0.89–1.37)	0.370	0.89 (0.75–1.07)	0.217
deCODE	547	26380	0.91 (0.80–1.03)	0.119	0.98 (0.82–1.17)	0.808	1.08 (0.92–1.28)	0.351
OncoArray	5161	11323	0.96 (0.91–1.00)	0.067	1.02 (0.95–1.09)	0.589	1.03 (0.98–1.10)	0.259
Overall	10023	54544	0.95 (0.91–0.98)	0.011	1.04 (0.99–1.09)	0.076	1.05 (1.00–1.12)	0.029
Squamous cell carcinoma			*I*^2^ = 0.0%, *P* = 0.857		*I*^2^ = 0.0%, *P* = 0.654		*I*^2^ = 0.0%, *P* = 0.599	
ICR	611	5200	0.94 (0.83–1.06)	0.339	1.13 (0.96–1.33)	0.146	1.08 (0.93–1.25)	0.300
MDACC	306	1134	1.05 (0.87–1.27)	0.630	1.17 (0.90–1.51)	0.246	1.14 (0.88–1.46)	0.317
IARC	911	2968	0.87 (0.78–0.97)	0.010	1.06 (0.92–1.22)	0.421	1.02 (0.89–1.18)	0.750
NCI	1447	5736	0.90 (0.83–0.98)	0.019	1.22 (1.09–1.36)	5.34E-04	1.12 (1.00–1.25)	0.040
Toronto	50	499	0.92 (0.58–1.47)	0.733	0.93 (0.50–1.76)	0.835	1.14 (0.65–2.03)	0.643
GLC	97	478	1.00 (0.72–1.38)	0.977	1.19 (0.77–1.83)	0.432	1.16 (0.77–1.76)	0.480
Harvard	216	970	0.84 (0.67–1.06)	0.142	0.86 (0.62–1.20)	0.383	1.35 (1.04–1.74)	0.023
deCODE	259	26380	0.92 (0.77–1.09)	0.335	1.07 (0.82–1.39)	0.618	0.91 (0.71–1.15)	0.426
OncoArray	3529	11323	0.91 (0.86–0.96)	3.00E−04	1.14 (1.06–1.22)	5.00E−04	1.06 (0.99–1.14)	0.073
Overall	7426	54688	0.91 (0.88–0.95)	1.81E−08	1.13 (1.08–1.19)	6.21E−08	1.08 (1.03–1.13)	7.93E−04
Ever smoking			*I*^2^ = 0.0%, *P* = 0.910		*I*^2^ = 0.0%, *P* = 0.675		*I*^2^ = 29.1%, *P* = 0.177	
IARC	2367	2508	0.95 (0.88-1.04)	0.274	1.11 (0.99-1.24)	0.068	1.12 (1.01-1.25)	0.037
Toronto	236	272	0.91 (0.68–1.21)	0.508	1.01 (0.69–1.49)	0.948	1.12 (0.79–1.58)	0.535
GLC	433	258	0.88 (0.69–1.14)	0.337	0.86 (0.62–1.18)	0.356	1.09 (0.80–1.49)	0.600
Harvard	892	809	0.95 (0.83–1.10)	0.504	1.11 (0.90–1.36)	0.333	0.99 (0.83–1.17)	0.870
MDACC	1150	1134	0.95 (0.84–1.07)	0.407	1.17 (0.99–1.39)	0.064	1.18 (1.00–1.40)	0.047
ATBC	1732	1270	0.95 (0.85–1.06)	0.339	1.14 (1.00–1.30)	0.055	1.03 (0.88–1.20)	0.693
CPSII	600	383	1.10 (0.90–1.34)	0.355	1.21 (0.92–1.59)	0.175	0.93 (0.74–1.18)	0.578
EAGLE	1767	1339	0.94 (0.84–1.04)	0.225	1.06 (0.91–1.22)	0.473	1.27 (1.13–1.43)	9.00E−05
PLCO	1243	1344	0.88 (0.78–0.99)	0.039	0.97 (0.83–1.15)	0.740	1.23 (1.06–1.43)	0.006
OncoArray	12803	7613	0.94 (0.90–0.98)	0.003	1.06 (1.01–1.12)	0.031	1.09 (1.04–1.15)	6.00E−04
Overall	23223	16930	0.94 (0.91–0.97)	9.80E-05	1.07 (1.03–1.12)	2.70E−04	1.12 (1.06–1.18)	2.90E−05
Never smoking			*I*^2^ = 0.0%, *P* = 0.700		*I*^2^ = 0.0%, *P* = 0.761		*I*^2^ = 0.0%, *P* = 0.939	
IARC	159	1253	0.87 (0.68–1.11)	0.253	1.08 (0.78–1.49)	0.647	1.09 (0.79–1.49)	0.602
Toronto	95	217	0.96 (0.65–1.42)	0.843	0.90 (0.50–1.61)	0.712	1.04 (0.66–1.64)	0.871
GLC	35	220	0.80 (0.47–1.36)	0.409	0.49 (0.19–1.26)	0.140	1.18 (0.58–2.39)	0.652
Harvard	92	161	0.86 (0.59–1.27)	0.461	1.07 (0.63–1.83)	0.803	0.86 (0.55–1.35)	0.520
CPSII	86	275	1.35 (0.92–1.97)	0.124	0.96 (0.53–1.73)	0.893	1.23 (0.77–1.97)	0.384
EAGLE	138	634	1.01 (0.77–1.34)	0.920	1.30 (0.87–1.93)	0.199	0.95 (0.68–1.33)	0.780
PLCO	126	470	1.01 (0.70–1.44)	0.975	1.18 (0.72–1.92)	0.513	1.08 (0.69–1.68)	0.735
OncoArray	1343	3463	0.96 (0.88–1.05)	0.397	1.07 (0.94–1.22)	0.282	0.96 (0.86–1.07)	0.409
Overall	2074	6693	0.96 (0.89–1.03)	0.215	1.07 (0.96–1.19)	0.155	0.98 (0.90–1.08)	0.892

Abbreviations: *GWAS* genome-wide association study, *AD* adenocarcinoma, *SC* squamous cell carcinoma, *OR* odds ratio, *CI* confidence interval, *I*^2^ heterogeneity statistic.

One of the major risk factors for lung cancer is cigarette smoking, which may interact with genetic factors. According to available smoking data, study subjects were divided into two groups as ever smokers and never smokers in stratified analyses. We found that rs329118, rs2285521, and rs198459 all had a significant risk effect in ever smokers (OR = 0.94, 95% CI = 0.91–0.97; OR = 1.07, 95% CI = 1.03–1.12; OR = 1.02, 95% CI = 1.06–1.18, respectively) (Table 2 and Supplementary Fig. 1), while no significant association was observed in never smokers for all three SNPs. The forest plots of the overall and stratification results for these three SNPs are shown in Supplementary Fig. 1a–c.

### In silico analyses

The three SNPs were not only predicted to potentially affect RNA splicing but also were predicted to potentially affect mRNA transcription (Supplementary Table 4). According to experiment-based data (e.g., histone modification, DNase cluster, transcription factor (TF) binding, RNAseq) from the ENCODE project (Fig. 2e, f), we found that two SNPs (*JADE2* rs329118 and *GGA2* rs2285521) are located within one CpG island with strong signals for active enhancer and promoter functions (indicated by H3K4 tri-methylation, histone modification H3K27 acetylation, and DNase hyper-sensitivity). *MYRF* rs198459 was also predicted to be located within a regulatory region with evidence for H3K4 mono-methylation and DNase cluster (Fig. 2g). Further TF binding analysis (using ChIP-seq data) showed that rs329118, rs2285521, and rs198459 are located within the AP2B, MYOD-1, and ELK3 motifs, respectively, as shown by the position weight matrix (PWM)-based Sequence Logo (Fig. 2e–g), which suggest that the allele difference might influence the binding activity of the TF.

In the following analyses using three mRNA expression and genotype databases, rs2285521 in *GGA2* was assessed directly or indirectly from the lymphoblastoid cell lines, normal lung tissue, and lung cancer tissue, while the other two variants rs329118 and rs198459 only had available data either from lymphoblastoid cell lines or whole blood cells. The rational for using different tissues was to test the potential tissue-specific regulation of these SNPs.

**Fig. 3:**
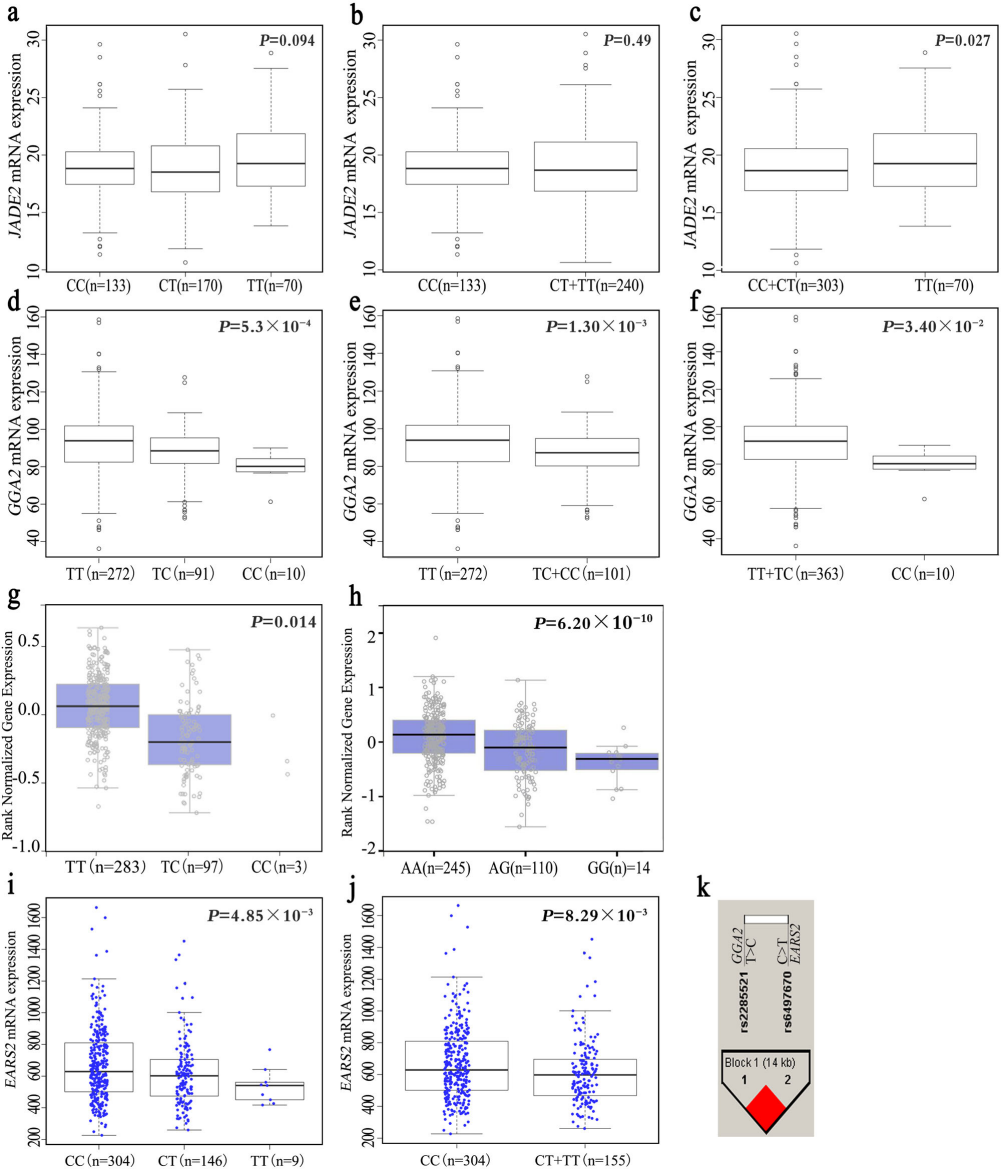
Functional analyses of rs329118 on *JADE2*, rs2285521 on *GGA2*, and rs198459 on *MYRF*. Correlation between *JADE2* rs329118 and *JADE2* mRNA expression levels in additive (**a**), dominant (**b**), and recessive (**c**) models in 373 blood cells from 373 Europeans individuals in 1000 genomes project (*P* = 0.094, 0.487 and 0.027, respectively). Correlation between *GGA2* rs2285521 and *GGA2* mRNA expression levels in additive (**d**), dominant (**e**), and recessive (**f**) models in 373 blood cells from 373 Europeans individuals in 1000 genomes project (*P* = 5.30 × 10^−4^, 0.0013 and 0.034, respectively). Correlation between *GGA2* rs2285521 (**g**) and *MYRF* rs198459 (**h**)*, and* mRNA expression levels in normal lung tissues or whole blood cells of GTEx project (*P* = 0.014 and *P* = 6.20 × 10^−10^, respectively). **i**, **j** Correlation between *EARS2* rs6497670 in additive (**i**) and dominant (**j**) models in lung cancer tissues of TCGA project (*P* = 4.85 × 10^−3^ and *P* = 8.29 × 10^−3^, respectively). **k** Pair-wise LD plot between *GGA2* rs2285521 (T>C) and *EARS2* rs6497670 (C>T). **a**–**j** *P* value was calculated from linear regression. Center-line indicates the median expression level across all participants in that group, and the hinges represent the lower (Q1) and upper (Q3) quartile, with lower whisker indicating the smallest value within 1.5 interquartile range (IQR) below Q1 and upper whisker indicating the largest value within 1.5 IQR above Q3.

Expression quantitative trait loci (eQTL) analysis was first performed to assess the association between each SNP and its target gene mRNA expression level in the lymphoblastoid cell lines from 373 subjects of European ancestry in the 1000 Genomes project. From this analysis, we found that the *JADE2* rs329118 T allele was significantly associated with increased expression levels of *JADE2* in a recessive model (*P* = 0.027), but not in an additive or dominant model (Fig. 3a–c). The *GGA2* rs2285521 C allele was significantly related to decreased mRNA expression levels in all additive (*P* = 5.30 × 10^−4^), dominant (*P* = 1.30 × 10^−3^), and recessive (*P* = 0.034) models (Fig. 3d–f).

SNP rs2285521 was also significantly correlated with mRNA expression levels of *GGA2* in normal lung tissues based on the Genotypes-Tissue Expression (GTEx) database (*P* = 0.014, Fig. 3g), which is consistent with the results in the lymphoblastoid cell lines. Similar results were found for *MYRF* rs198459 in whole blood cells (*P* = 6.20 × 10^−10^) (Fig. 3h). Using both genotyping and gene expression data for lung cancer in the Cancer Genome Atlas (TCGA) database, we performed SNP-mRNA correlation analyses for the three SNPs. However, we were only able to retrieve the genotype data for rs6497670 in *EASR2* (Fig. 3i, j), a SNP in complete linkage disequilibrium (LD) (*r*^2^ = 1) with rs2285521 in *GGA2*, with available data after performing imputation for these top three SNPs (Fig. 3k). Once again, SNP rs2285521 showed a significant correlation with decreased mRNA expression levels of *GGA2* in lung cancer tissues, specifically in both additive (*P* = 4.85 × 10^−3^, Fig. 3i) and recessive (*P* = 8.29 × 10^−3^, Fig. 3j) models for LUSC, but not for LUAD (Supplementary Fig. 2e, f).

Results from other studies collected in the cancer microarray database Oncomine also provided evidence for a higher expression level of JADE2 in NSCLC tissue (both LUAD and LUSC) than in normal lung tissue. Both GGA2 and MYRF were found to exhibit lower expression in NSCLC (both LUAD and LUSC) tissue than in normal lung tissue (Supplementary Fig. 3a–f).

**Fig. 4:**
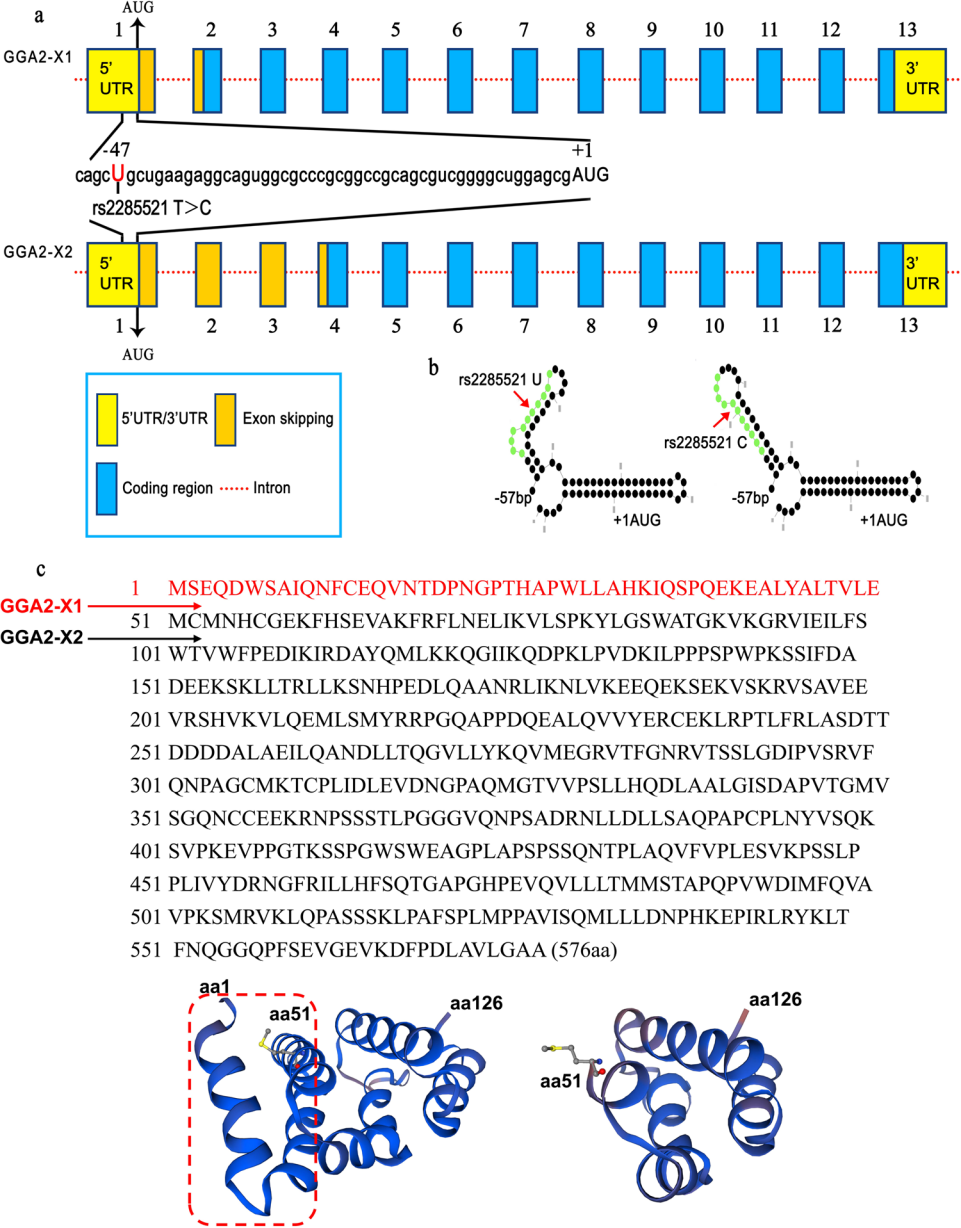
Diagram representation of the alternative splicing pattern of *GGA2* transcripts and the amino acid (aa) sequence, protein structures, and domains of the GGA2-isoforms. **a** Results of SNP rs2285521 sequencing in the 53 bp sequence of 5’ UTR region in Exon 1 of the *GGA2*: Genomic structure of the *GGA2-X1 and GGA2-X2*. Splicing pattern of the *GGA2* variant with SNP rs2285521 T>C. The position of the A nucleotide in the start codon (ATG) is defined as +1. **b** Part of RNA secondary structure of the *GGA2* rs2285521 U *and GGA2* rs2285521 C. **c** The aa sequence of GGA2-X1 and GGA2-X2. The GGA2-X1 isoform encodes a 576 aa protein and GGA2-X2 encodes a 526 aa protein. The aa sequence of red is where GGA2-X2 starts. **d** Part of the secondary structures and domains of putative isoforms of GGA2-X1 and GGA2-X2. aa1-aa50 circled by red was the part two isoforms differed.

For *GGA2*, we compared two *GGA2* transcripts (GenBank number: NC_000016) (Fig. 4a–d), which were designated as *GGA2-X1* and *GGA2-X2* in the GenBank of National Center of Biotechnology Information. We analyzed the amino acid (aa) sequence and secondary structure of the putative protein isoforms of GGA2-X1 and GGA2-X2 with ExPASy and SWISS-MODEL, respectively. The aa sequence (Fig. 4a) and secondary structure differed between the two isoforms (Fig. 4b). The GGA2-X1 isoform contained 576 aa, while the GGA2-X2 isoform contained 526 aa. There is complete homology between GGA2-X1 and GGA2-X2 in the last nine exons of C-terminal sequences, but GGA2-X2 lacks 50 aa (aa1-aa50) in the N-terminal sequences, compared with GGA2-X1, as shown in Fig. 4c, d.

## Discussion

To our knowledge, we are the first to report a genome-wide profiling analysis of RNA splicing-related genetic variants in lung cancer, using a total of 29,266 cases and 56,450 non-cancer controls of European ancestry. We identified three genetic variants (rs329118, rs198459, and rs2285521) to be associated with lung cancer risk, residing in a gene for apoptosis and differentiation in epithelia 2 (JADE family PHD zinc finger 2; *JADE2*; PHF16) at 5q31.1, myelin regulatory factor gene (*MYRF*) at 11q12.2, and golgi-localized, γ-adaptin ear-containing, ADP ribosylation factor-binding protein 2 (*GGA2*) at 16p12.2, respectively.

Among the three identified susceptibility genes in the present study, *GGA2* belongs to the GGA family, which consists of three isoforms in vivo, namely *GGA1*, *GGA2*, and *GGA3*^22^. These isoforms have been reported to have transport functions in cells, with GGA2 predominantly in the trans-Golgi network (TGN) and endoplasmic reticulum, participating in the separation of the TGN and polyvesicles^22,23^. GGA2 cooperates with mannose 6-phosphate receptor and adaptor protein-1 in regulating protein sorting, showing significant co-localization with transporter glucose transporter 4 (GLUT4) recovery pool^24^. It also stimulates the activation of EGFR signal transduction and promotes the occurrence and development of several malignancies^22,25^. SNP rs2285521 T>C is located in the first exon of *GGA2* on chr16, which lies upstream of the coding sequence, 48 bp away from the translation initiation site, belonging to the 5 ‘untranslated region (UTR). It appears that rs2285521 T>C does not directly change the sequence or the amino acid types of GGA2, but the mRNA structure analysis suggests that the conversion of rs2285521 allele T to C changes a loop-stem-loop secondary structure. In the GenBank of National Center of Biotechnology Information, there are two isoforms of GGA2: GGA2-X1 and GGA2-X2, generated by an exon skipping/inclusion event. Compared with GGA2-X1, GGA2-X2 lacks 50 amino acids (aa1–aa50) in the N-terminal sequences. The exon skipping/inclusion event leads to a difference in the N-terminal sequences between GGA2-X1 and GGA2-X2, while keeping the complete homology of the two isoforms in the remaining nine exons of C-terminal sequences. Whether rs2285521 is involved in the regulation of this exon skipping/inclusion event remains to be determined. Meanwhile, both the eQTL data from lymphocytes and GTEx data from lung tissue suggest that rs2285521 is involved in transcriptional regulation possibly by reducing the mRNA expression of *GGA2*. In addition, TCGA data showed that the substitution of the T allele with the C allele would lead to a decrease in *GGA2* mRNA expression in LUSC. The in silico results of these three analyses supported the observed genetic associations. These data also suggest that different histological subtypes of lung cancer may have different genetic etiology, with *GGA2* rs2285521 more likely having significance in LUSC.

Two other variants, rs329118 and rs198459 are located in the introns of *JADE2* and *MYRF*, respectively. JADE2 is a member of the small JADE family that also includes JADE1 (PHF17) and JADE3 (PHF15) paralogs^26^. All three JADE proteins bear in tandem two Plant Homeo-domains (PHD), which are zinc finger domains^26^. Jade-1 was reported to inhibit Wnt signaling through its E3 ubiquitin ligase activity of β-catenin and was therefore defined as a PHD-finger-type E3 ubiquitin ligase^27^. *JADE2* rs329118 was recently shown to be associated with age of initiation of regular smoking, but no relation to lung cancer was reported^28^. So far, it is known that *JADE1* mRNA encodes two protein products: the full length isoform JADE1L and the truncated isoform JADE1S as a result of alternative RNA splicing^26^, but the RNA splicing pattern of *JADE2, as well as its molecular function,* remain unclear.

*MYRF* encodes an endoplasmic reticulum membrane protein that undergoes auto-processing to release its N-terminal fragment, which enters the nucleus, forms a homo-trimer, and functions as a TF^29^. There is an increasing evidence that *MYRF* may play a critical role in the development of various organs, including the heart, lungs, diaphragm, and genitourinary tract. For example, particular missense or frameshift variants in *MYRF* have been identified to be linked to mild encephalitis/encephalopathy or congenital heart defects, and/or congenital diaphragmatic hernia^30^. Interestingly, members of our team have recently reported that *MYRF* is a differentially spliced gene in LUSC between patients of West African and European ancestry who are smokers^31^.

In summary, the present study, with the largest lung cancer study population of European ancestry ever reported, identified three genetic variants in genome-wide profiling RNA splicing-related genes to be associated with lung cancer risk. We believed that all susceptibility alleles, if biologically meaningful, should be correlated with expression levels of the corresponding genes in normal lymphocytes and/or lung tissues. Our results suggest that the combination of genetics and in silico analyses helps identify and emphasize potential functional importance of RNA splicing-related loci in lung cancer susceptibility, providing insights into the etiology of this complex disease. However, replication of the results is also required in different populations as well in larger prospective studies. Because the underlying molecular splicing mechanisms of the RNA splicing-related genetic variants in lung cancer are not completely understood, further biological validation both in vitro and in vivo are warranted in the future to better understand the role of these three SNPs.

## Methods

### Study design and subjects

The present study adopted a two-stage design with discovery and replication datasets. The discovery set was comprised of 14,463 lung cancer cases and 44,188 non-cancer controls of European ancestry from eight centers. The replication series was comprised of 14,803 cases and non-cancer 12,262 controls of European ancestry from 31 sites, of which some centers (with no overlapping study subjects) also participated in the discovery phase (Supplementary Table 1).

### Discovery set

The study populations of the discovery set have been described in previous publications from TRICL-ILCCO^20,32^. Briefly, eight published lung cancer GWASs were from the TRICL-ILCCO consortia, which consists of 14,463 lung cancer cases and 44,188 controls of European ancestry. The GWAS participants included Institute of Cancer Research (ICR), The University of Texas MD Anderson Cancer Center (MDACC), International Agency for Research on Cancer (IARC), National Cancer Institute (NCI), Lunenfeld-Tanenbaum Research Institute study (Toronto), German Lung Cancer Study (GLC), the Harvard Lung Cancer Study, and Icelandic Lung Cancer Study (deCODE)^20,33^. (Supplementary Table 1)

### Replication set

The replication series was comprised of 14,803 cases and 12,262 controls from 31 study sites, of which some centers (but not study subjects) also participated in the discovery phase. Comprehensive details of each series have been previously reported^16,21,34–36^. After excluding samples genetically identified as overlapping between the OncoArray and the TRICL-ILCCO, 14,463 cases and 44,188 controls from the discovery set and 14,803 cases and 12,262 controls from the OncoArray were included in the final analyses. Most of the lung cancer cases had been histologically confirmed to be lung adenocarcinoma, followed by LUSC, and lung small cell carcinoma. Given distinct differences in smoking status and histological subtypes, the subgroup analyses, including ever and never-smokers as well as lung adenocarcinomas and LUSC, were performed. All ever smokers in the present study were defined as individuals having smoked at least 100 cigarettes in their lifetime and never-smokers defined as individuals who had smoked less than 100 cigarettes during their lifetime. A written informed consent was obtained from each participant of each dataset. The present study was approved by the Duke University Health System Institutional Review Board, and all methods performed in the present study were in accordance with the relevant guidelines and regulations.

### Genotyping and quality control

For all of the GWAS datasets in TRICL-ILCCO, multiple genotyping platforms were applied, including Illumina HumanHap 317, 317 + 240S, 370Duo, 550, 610, or 1M arrays^37^. For the meta-analyses, imputation was performed based on the reference data from the 1000 Genomes Project (phase I integrated release 3, March 2012) by using both IMPUTE2 v2.1.1^38^ and MaCH v1.0 + minimac (version 2012.10.3) softwares^39^. Only SNPs with an information score ≥0.40 in IMPUTE2 or an *r*^2^ ≥ 0.30 in MaCH were included in the final analyses. Standard quality control on samples was performed on all scans, excluding individuals with a low call rate (<90%), extremely high or low heterozygosity (*P* < 1.0 × 10^−4^), and non-European ancestry (using the HapMap phase II CEU, JPT/CHB, and YRI populations as reference).

The OncoArray consortium genotyping was completed at the Center for Inherited Disease Research (CIDR), the Helmholtz Center Munich (HMGU), Copenhagen University Hospital, and the University of Cambridge. The quality control procedures for the OncoArray dataset were identical and are reported elsewhere^16,21,36^. Briefly, genotype definition was undertaken using Genome Studio and jointly clustered data from 57,775 individuals and 533,631 SNPs. This included 44,591 samples associated with this study of lung cancer, 12,901 individuals from other unrelated OncoArray studies, and 283 HapMap control individuals of European, African, Chinese, and Japanese origin. Among 44,591 OncoArray lung cancer samples, 17,526 samples, including 1193 QC duplicate samples, 7633 samples overlapped with the discovery sets, 1708 with low call rate less than 95%, 1280 samples with PI-HAT greater than 0.95 or between 0.45 and 0.95 in IBD analysis, 306 with sex inconsistency information, and 5407 individuals with non-Caucasian ancestry were removed from the OncoArray when performing the validation OncoArray analysis and the joint analysis of the discovery and OncoArray sets. A total of 27,065 OncoArray samples were included in final association analysis including 14,803 lung cancer cases and 12,262 controls. Additionally, 4348 samples genotyped on the OncoArray and in a prior study including 1926 from MDACC, 2422 from IARC, and 9,811 samples without linked disease information used for genotype clustering were removed from the OncoArray set in the joint analysis of the discovery and OncoArray sets. Finally, 25,978 samples remained. The OncoArray genotyping platform queried 533,631 SNPs for fine mapping of lung cancer susceptibility loci as well as for additional de novo discovery. We used OncoArray samples for a validation of six top variants from TRICL-ILCCO samples, and we performed a meta-analysis of the discovery set and the validation set.

### SNP selection

All SNPs were selected from both the 1000 Genomes project^40^ and GTEx Portal project (383 normal lung tissues)^41^. By using the genotyping data from the 1000 Genomes project, we filtered out SNPs with a minor allele frequency (MAF) < 0.01 in populations of European ancestry. After that, there remained 9,739,729 SNPs with MAF ≥ 0.01 in Caucasian populations. We then performed functional prediction for these SNPs using SNPinfo software^42^ and the Ensembl Variant Effect Predictor (VEP) software^43^, which integrated the RNA splicing prediction algorithms of FAS–ESS, RESCUE ESE, ESEfinder, MaxEntScan, Ada Boost, and Random Forest in dbscSNV^44^. The algorithm of SNPinfo uses GWAS SNP *P*-value data and finds all SNPs in high LD with GWAS SNPs, so that selection is from a much larger set of SNPs than the GWAS itself^41^. The Ensembl VEP software provides tools and methods for a systematic approach to annotate and prioritize variants in both large-scale sequencing projects and smaller analysis studies^42^. By using SNPinfo, we found 36,142 predicted RNA splicing-related SNPs in Caucasian populations. Through the Ensembl VEP, we identified 16,306 potential RNA splicing-related SNPs. After combining all SNPs, there were 48,305 non-redundant SNPs in Caucasian populations. Meanwhile, 92,213 and 837,755 potential RNA splicing-related SNPs with MAF ≥ 0.01 were predicted from the GTEx Portal project by sQTLseekeR package and Altrans method, respectively^45,46^. sQTLseekeR is an R package to identify splicing quantitative trait loci (sQTL) in transcriptome population studies. It can be downloaded from http://big.crg.cat/computational_biology_of_rna_processing/sqtlseeker. sQTLseekeR could be directly employed for joint analysis of gene expression across tissues. it could also be used to identify SNPs affecting expression networks, where the multivariate phenotype is the relative expression of gene compared with the total expression output of the network^44^. Altrans is another method for discovery of alternative sQTLs^45^. In searching for alternative sQTLs, nearly all methods have to infer quantifications of transcripts or splice junctions, and each method has its relative merits. However, Altrans is capable of identifying thousands of sQTLs, many of which are missed by other methods. It is very sensitive and performs comparably to other methods^45^. Because each of these tools has its own unique algorithm and merits, we expected some variation in the final results generated by these tools.

After comparing the final SNP sets from the two projects mentioned above, a total of 5,182 mutual SNPs with *P*-value < 0.05 were retained. The *P*-values were extracted from the sQTL results of lung tissues of the GTEx project. More details about the sQTL analysis could be found in the previous GTEx publication^40^. Briefly, two complementary approaches (sQTLseekeR^44^ and Altrans^45^) were used to quantify the relative expression of splicing isoforms. For sQTLseekeR, the association between SNPs with ±5 kb of the corresponding gene and the splicing ratios of gene transcript isoforms by using a non-parametrical approach: a F score was calculated by comparing the variability of splicing ratios within genotypes with the variability between genotypes, after which permutation was performed to estimate the significance of the score and calculate *P* value. For Altrans, the association between SNPs within ±1 Mb region around transcription start sites and the expression levels of exon junctions were tested by using the Spearman’s rank correlation test with adjustment for the first three principal components. There were finally a total of 4482 SNPs extracted in the TRICL-ILLCO dataset. Additional details are shown in Fig. 1.

### In silico functional analyses

Based on the association results of genetic variants and lung cancer risk, bioinformatic analyses for functional prediction were performed on the top three significant SNPs, in particular their potential ability to affect mRNA splicing function and transcription. Four in silico tools: F-SNP^47^, SNPinfo Web Server^42^, RegulomeDB^48^, and HaploReg^49^ were selected for further predictive functional analyses of the significant SNPs. We then performed an eQTL analysis to assess the association between SNPs and mRNA expression level of the corresponding gene using the mRNA expression data from the lymphoblastoid cell lines of 373 subjects of European ancestry available in the 1000 Genomes Project^40^ and the eQTL results from the GTEx project^41^ as well as 127 NSCLC tissues in TCGA^50,51^. If no direct genotyping data for the three SNPs could be retrieved in the databases, then all other SNPs, which were in complete LD (*r*^2^ = 1) with the corresponding SNP generated by the imputation were also checked. The available data of those SNPs would then be retrieved and analyzed. In addition, we compared the mRNA expression levels of target genes between NSCLC and adjacent normal tissues available in the Oncomine^TM^ database^52^. To explore the potential regulation mechanism of the aberrant splicing, possible allelic effects of these variants on TF-binding motifs were determined using PrEdict Regulatory Functional Effect of SNPs by Approximate *P* value Estimation (PERFECTOS-APE; http://opera.autosome.ru/perfectosape/), which determines the probability of a TF motif (using position weight matrices, from HOCOMOCO-10, JASPAR, HTSELEX, SwissRegulon, and HOMER databases) in the DNA sequence overlapping each variant. The fold change in the probability of a TF binding site present for each allele of a variant was then calculated^53^.

### Analysis and prediction of GGA2 splicing variants

The secondary RNA structure of human GGA2 splicing variants was predicted with RNA-Folding-Form^54^. The secondary protein structure of human GGA2 splicing variants was predicted with SWISS-MODEL^55^.

### Statistical methods

We performed an unconditional logistic regression to estimate ORs and 95% CIs per effect allele for each GWAS dataset using R (v2.6), Stata (v10, State College, TX, USA), and PLINK (v1.06) software with adjustment for the top significant principal components^32^. We used a fixed effects model to perform meta-analysis by the inverse variance method^56^. If the Cochran’s Q test *P*-value ≤ 0.100 or the heterogeneity statistic (*I*^2^) ≥ 25%, a random-effects model was employed. We used the linear step-up method of Benjamini and Hochberg to calculate FDR with a cut-off value of 0.20 to correct for multiple comparisons^57^ and used linear regression for the eQTL analysis and paired t-test for the gene differential expression analysis between tumor and adjacent normal tissues. Based on the 1000 Genomes Phase I integrated variant set (release 201203), we used Haploview v4.2^58^ to construct the LD plots, respectively. SNP pruning was applied, and SNPs with pair-wise *r*^2^ < 0.30 were considered as independent. All other analyses were conducted with SAS (version 9.4; SAS Institute, Cary, NC, USA), if not specified otherwise.

### Reporting summary

Further information on research design is available in the Nature Research Reporting Summary linked to this article.

## Supplementary information

Supplementary data

Reporting Summary

## Data Availability

Selected data that support the findings of this study are available from the corresponding author upon reasonable request and ccorrespondence should be addressed to S.R.P. or Q.W. The primary and processed data used to generate the analyses presented here are available in the following locations: The phase1 release of variant calls of the 1000 Genomes Project^39^ was downloaded from https://mathgen.stats.ox.ac.uk/impute/impute_v2.html. The data deposited in and available from the GTEx Portal^40^ can be downloaded from www.gtexportal.org. Some other data that support the findings of this study are available via the dbGaP (www.ncbi.nlm.nih.gov/gap, database of genotypes and phenotypes) repository, among which the data are controlled-access, so interested readers will need to request access. The information on how to do so can be found on pages linked to below. The access numbers are https://identifiers.org/dbgap:phs000876.v2.p1^59^ for TRICL study, https://identifiers.org/dbgap:phs001273.v3.p2^60^ for the OncoArray study, and https://identifiers.org/dbgap:phs000178.v11.p8^61^ for TCGA study.

## Code availability

We used SAS (version 9.4; SAS Institute, Cary, NC, USA), R (v2.6), Stata (v10, State College, TX, USA), and PLINK (v1.06) softwares for data analysis. Only standard scripts were used for this study. No custom codes or mathematical algorithms were applied.
