# Supplementary material for: Deciphering associations between three RNA splicing-related genetic variants and lung cancer risk
Source: NPJ Precis Oncol. 2022 Jun 30;6:48. doi: 10.1038/s41698-022-00281-9 (PMC9247007; doi:10.1038/s41698-022-00281-9)

Supplementary Table 1. Characteristics of the study populations included in the participating TRICL-ILLCO and OncoArray datasets

Study	Overall		Histological types				Smoking status			
			LUAD		LUSC		Never smoking		Ever smoking	
	Cases	Controls	Cases	Controls	Cases	Controls	Cases	Controls	Cases	Controls
**TRICL Total**	14463	44188	4862	43221	3897	43365	731	3230	10420	9317
ICR[1]	1952	5200	465	5200	611	5200	-	-	-	-
MDACC[2]	1150	1134	619	1134	306	1134	-	-	1150	1134
IARC[3]	2533	3791	517	2824	911	2968	159	1253	2367	2508
NCI[4]	5713	5736	1841	5736	1447	5736	350	1379	5342	4336
Toronto[5]	331	499	90	499	50	499	95	217	236	272
GLC[6]	481	478	186	478	97	478	35	220	433	258
Harvard[7]	984	970	597	970	216	970	92	161	892	809
Decode[8]	1319	26380	547	26380	259	26380	-	-	-	-
**OncoArray**	14803	12262	6411	12262	3529	12262	1624	4274	12803	7647
**Total**	29266	56450	11273	55483	7426	55627	2355	7504	23223	16964

Abbreviations: LUAD, lung adenocarcinoma; LUSC, lung squamous cell carcinoma.

[1] ICR: the Institute of Cancer Research Genome-wide Association Study, UK;

[2] MDACC: the MD Anderson Cancer Center Genome-wide Association Study, US;

[3] IARC: the International Agency for Research on Cancer Genome-wide Association Study, France;

[4] NCI: the National Cancer Institute Genome-wide Association Study, US; this study was composed of four GWASs: the Alpha-Tocopherol, Beta-Carotene Cancer Prevention Study (ATBC), the Cancer Prevention Study II Nutrition Cohort (CPSII), the Environment and Genetics in Lung Cancer Etiology (EAGLE) and the Prostate, Lung, Colon, Ovary Screening Trial (PLCO).

[5] Toronto: the Lunenfeld-Tanenbaum Research Institute Genome-wide Association Study, Toronto, Canada;

[6] GLC: German Lung Cancer Study, Germany;

[7] Harvard: Harvard Lung Cancer Study, US;

[8] deCODE: Icelandic Lung Cancer Study, Iceland

Supplementary Table 2. Discovery of SNPs associated with lung cancer risk in TRICL-ILLCO with FDR < 0.2

SNP rs#	Chr	Position	Alleles[a]	MAF	Encode gene	Effects	OR (95%CI)[b]	*P*[b]	FDR	P_{hete}	I^2	Reason for exclusion[c]
rs2276631	2	219249013	T/C	0.26	*SLC11A1*	-----+	0.93 (0.90-0.97)	4.72E-04	0.159	0.508	0	--
rs58309239	4	25443366	G/T	0.05	*LOC105374536*	------	0.85 (0.78-0.93)	1.77E-04	0.079	0.714	0	--
rs329118	5	133861663	T/C	0.42	*JADE2*	------	0.93 (0.90-0.96)	5.03E-05	0.032	0.983	0	--
rs115037049[d]	6	29977145	C/G	0.28	*HLA-J*	+++++-	1.11 (1.06-1.17)	4.96E-05	0.032	0.205	30.7	2
rs3095298	6	31082932	T/C	0.21	*PSORS1C1*	++++-+	1.09 (1.05-1.14)	3.07E-05	0.027	0.912	0	2
rs3131003	6	31093482	A/G	0.43	*PSORS1C1*	------	0.93 (0.90-0.97)	1.04E-04	0.058	0.41	0.9	2
rs707937	6	31731014	G/C	0.18	*SAPCD1*	--+---	0.91 (0.87-0.96)	2.30E-04	0.094	0.234	26.8	2
rs1150757	6	32029205	A/G	0.1	*TNXB*	++++++	1.20 (1.13-1.27)	2.88E-10	6.45E-07	0.254	24	2
rs6584410	10	102783678	T/C	0.42	*PDZD7*	------	0.93 (0.90-0.97)	3.72E-04	0.139	0.935	0	1
rs198459	11	61525020	A/G	0.22	*MYRF*	++++++	1.11 (1.06-1.16)	2.71E-06	0.003	0.491	0	--
rs3184504	12	111884608	T/C	0.48	*SH2B3*	------	0.93 (0.90-0.97)	1.75E-04	0.079	0.577	0	--
rs1898883	15	40655845	C/G	0.33	*DISP2*	------	0.94 (0.90-0.97)	4.98E-04	0.159	0.992	0	2
rs2127898	15	79083120	A/G	0.24	*ADAMTS7*	------	0.90 (0.86-0.94)	9.70E-07	1.45E-03	0.688	0	2
rs2285521	16	23521780	C/T	0.16	*GGA2*	++++-+	1.09 (1.04-1.14)	5.90E-04	0.165	0.749	0	--

Abbreviations: SNP, single nucleotide polymorphism; Chr, Chromosome; MAF, minor allele frequency; OR, odds ratio; CI, confidence interval; FDR, false discovery rate; hete, heterogeneity test.

a. Effect allele/Reference allele.

b. Adjusted for top principle components.

c. Genetic variant was previously reported to be associated with lung cancer risk (1) or existed linkage disequilibrium (r^2>0.1, D'=1) with previous reported SNP associated with lung cancer risk according to data obtained from Pubmed (2).

d. The new dbSNP ID of rs115037049 is rs356969.

Supplementary Table 3. the overall imputation info/RSQ of the SNPs in the eight TRICL GWASs

rs_number	info_s1	RSQ_s2	RSQ_s3	info_s4	info_s5	rsq_s6	rsq_s7	info_s8
rs329118	0.95636	0.951	0.9556	0.9729	0.95862	0.958	0.99707	0.96473
rs2285521	0.99161	0.9965	0.9985	0.9978	0.99063	0.999	0.95665	0.9924
rs198459	0.9185	0.77981	0.819	0.9228	0.88282	0.866	0.90815	0.89575

Abbreviations: info, information; RSQ, R-squared.

Supplementary Table 4. Functional prediction of three SNPs

SNP rs#	Chr	Gene	Aliases gene(s)	Location	SNPinfo[a]	Splicing function[a]	RegulomeDB score[b]	HaploReg v4.1[c]					
								Promoter histone marks	Enhancer histone marks	DNase	Proteins bound	Motifs changed	eQTL hit(s)
rs329118	5q31.1	*JADE2*	PFH15	133bp 5' of PHF15	TFBS	Y	4	24 tissues	33 tissues		6 bound proteins	Rad21, SP1, Sin3Ak-20	‘--
rs2285521	16p12.2	*GGA2*	VEAR	5’ UTR	TFBS	Y	4	24 tissues	53 tissues		42 bound proteins	5 altered motifs	62
rs198459	11q12.2	*MYRF*	C11orf9, MRF	Intron		Y	5	4 tissues	12 tissues	4tissues		GR, Irx	10

[a] https://snpinfo.niehs.nih.gov/cgi-bin/snpinfo/snpfunc.cgi; [b] http://www.regulomedb.org/index; [c] https://pubs.broadinstitute.org/mammals/haploreg/haploreg.php

Supplementary Figure 1. **(a)** Forest plots of rs329118; **(b)** rs2285521; **(c)** rs198459, which present the overall and stratification results of the three SNPs by histological types (adenocarcinoma and squamous carcinoma) and smoking status (ever and never). Note, in the stratification result by smoking status, the NCI study was composed of four sub-studies: the Alpha-Tocopherol, Beta-Carotene Cancer Prevention Study (ATBC), the Cancer Prevention Study II Nutrition Cohort (CPS-II), the Environment and Genetics in Lung Cancer Etiology (EAGLE), and the Prostate, Lung, Colon, Ovary Screening Trial (PLCO).

a

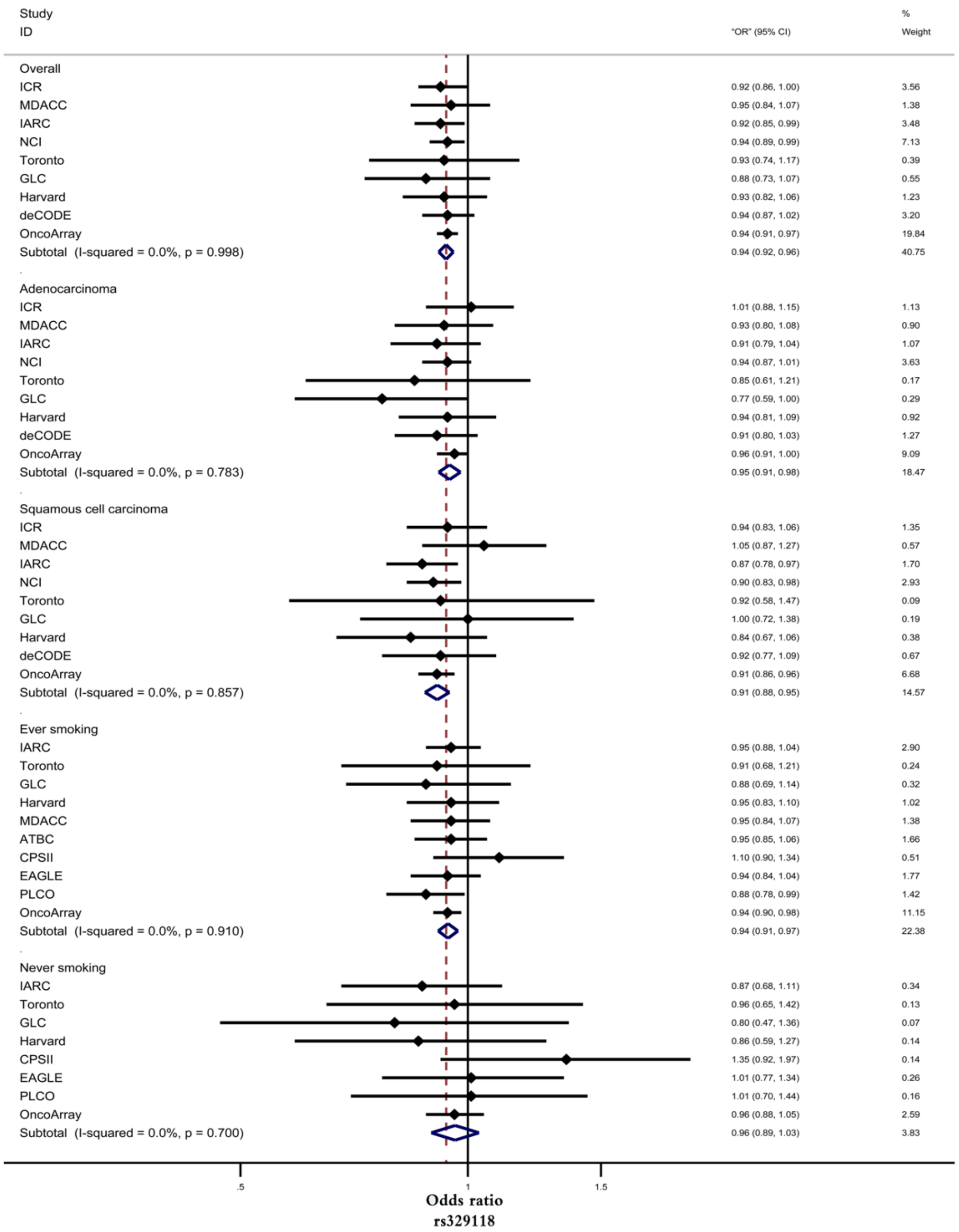

b

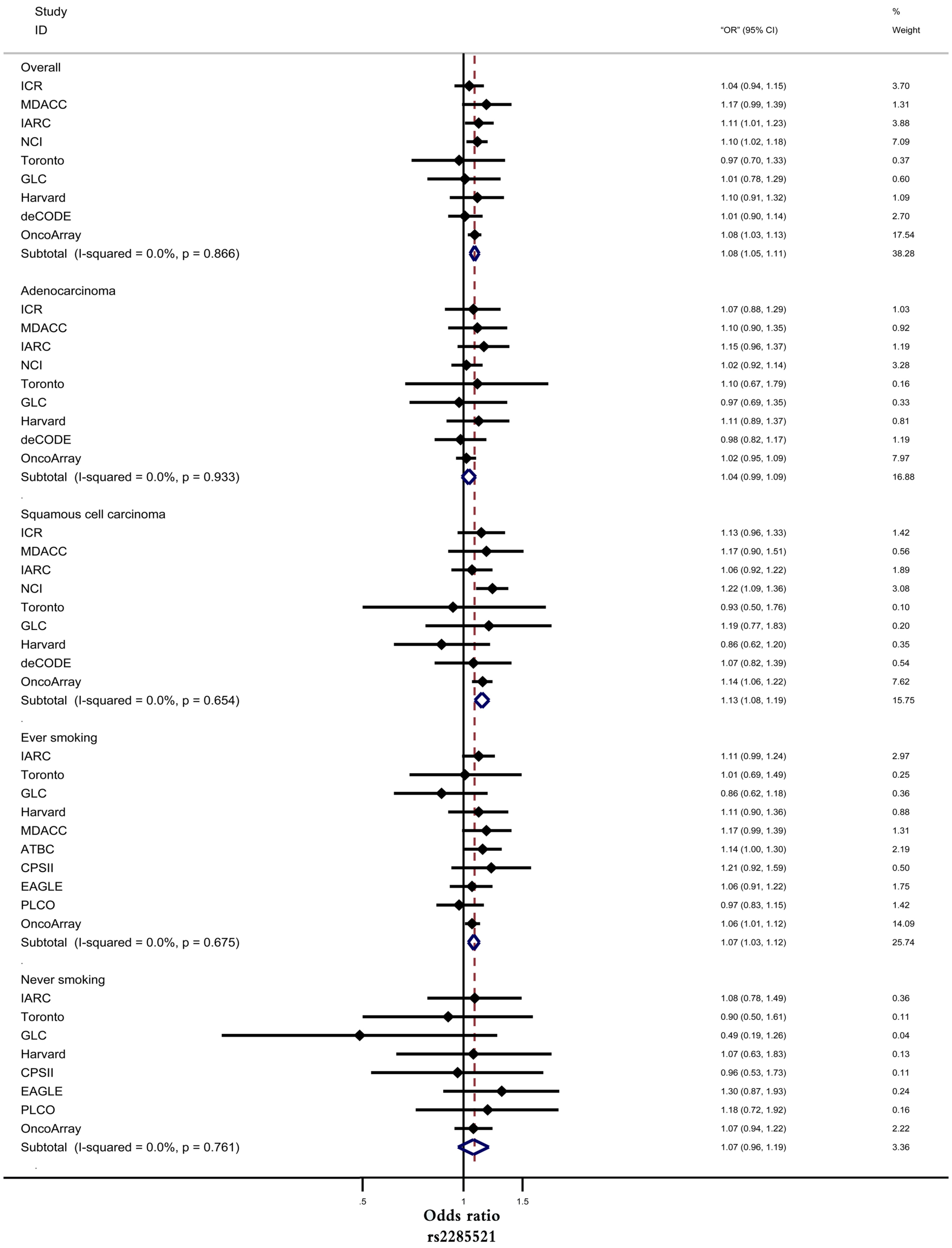
Study
ID
"OR" (95% CI)
%
Weight
Overall
ICR 1.04 (0.94, 1.15) 3.70
MDACC 1.17 (0.99, 1.39) 1.31
IARC 1.11 (1.01, 1.23) 3.88
NCI 1.10 (1.02, 1.18) 7.09
Toronto 0.97 (0.70, 1.33) 0.37
GLC 1.01 (0.78, 1.29) 0.60
Harvard 1.10 (0.91, 1.32) 1.09
deCODE 1.01 (0.90, 1.14) 2.70
OncoArray 1.08 (1.03, 1.13) 17.54
Subtotal (I-squared = 0.0%, p = 0.866) 1.08 (1.05, 1.11) 38.28
Adenocarcinoma
ICR 1.07 (0.88, 1.29) 1.03
MDACC 1.10 (0.90, 1.35) 0.92
IARC 1.15 (0.96, 1.37) 1.19
NCI 1.02 (0.92, 1.14) 3.28
Toronto 1.10 (0.67, 1.79) 0.16
GLC 0.97 (0.69, 1.35) 0.33
Harvard 1.11 (0.89, 1.37) 0.81
deCODE 0.98 (0.82, 1.17) 1.19
OncoArray 1.02 (0.95, 1.09) 7.97
Subtotal (I-squared = 0.0%, p = 0.933) 1.04 (0.99, 1.09) 16.88
Squamous cell carcinoma
ICR 1.13 (0.96, 1.33) 1.42
MDACC 1.17 (0.90, 1.51) 0.56
IARC 1.06 (0.92, 1.22) 1.89
NCI 1.22 (1.09, 1.36) 3.08
Toronto 0.93 (0.50, 1.76) 0.10
GLC 1.19 (0.77, 1.83) 0.20
Harvard 0.86 (0.62, 1.20) 0.35
deCODE 1.07 (0.82, 1.39) 0.54
OncoArray 1.14 (1.06, 1.22) 7.62
Subtotal (I-squared = 0.0%, p = 0.654) 1.13 (1.08, 1.19) 15.75
Ever smoking
IARC 1.11 (0.99, 1.24) 2.97
Toronto 1.01 (0.69, 1.49) 0.25
GLC 0.86 (0.62, 1.18) 0.36
Harvard 1.11 (0.90, 1.36) 0.88
MDACC 1.17 (0.99, 1.39) 1.31
ATBC 1.14 (1.00, 1.30) 2.19
CPSII 1.21 (0.92, 1.59) 0.50
EAGLE 1.06 (0.91, 1.22) 1.75
PLCO 0.97 (0.83, 1.15) 1.42
OncoArray 1.06 (1.01, 1.12) 14.09
Subtotal (I-squared = 0.0%, p = 0.675) 1.07 (1.03, 1.12) 25.74
Never smoking
IARC 1.08 (0.78, 1.49) 0.36
Toronto 0.90 (0.50, 1.61) 0.11
GLC 0.49 (0.19, 1.26) 0.04
Harvard 1.07 (0.63, 1.83) 0.13
CPSII 0.96 (0.53, 1.73) 0.11
EAGLE 1.30 (0.87, 1.93) 0.24
PLCO 1.18 (0.72, 1.92) 0.16
OncoArray 1.07 (0.94, 1.22) 2.22
Subtotal (I-squared = 0.0%, p = 0.761) 1.07 (0.96, 1.19) 3.36
.5
1
1.5
Odds ratio
rs2285521

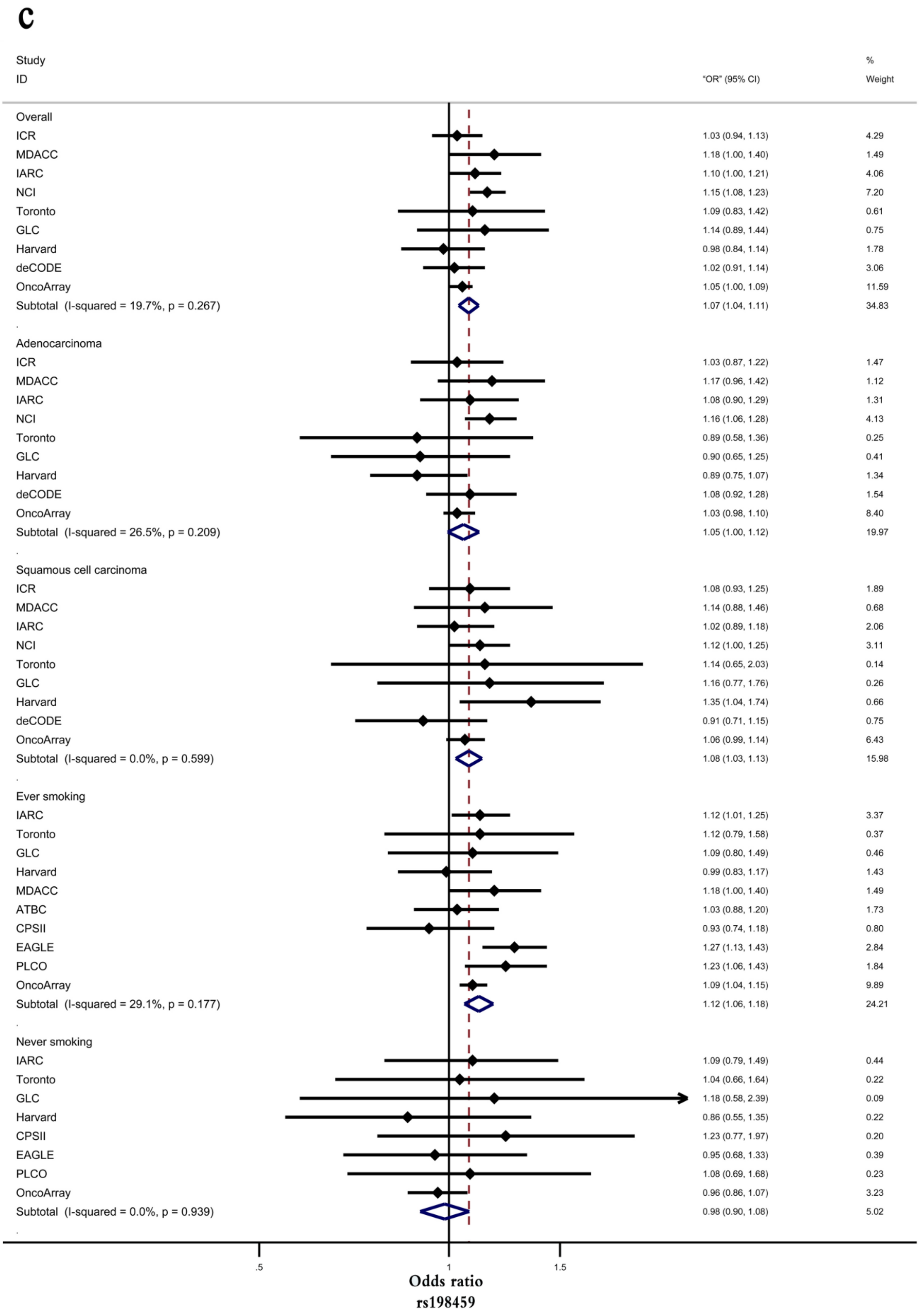
c
Study
ID
"OR" (95% CI)
%
Weight
Overall
ICR 1.03 (0.94, 1.13) 4.29
MDACC 1.18 (1.00, 1.40) 1.49
IARC 1.10 (1.00, 1.21) 4.06
NCI 1.15 (1.08, 1.23) 7.20
Toronto 1.09 (0.83, 1.42) 0.61
GLC 1.14 (0.89, 1.44) 0.75
Harvard 0.98 (0.84, 1.14) 1.78
deCODE 1.02 (0.91, 1.14) 3.06
OncoArray 1.05 (1.00, 1.09) 11.59
Subtotal (I-squared = 19.7%, p = 0.267) 1.07 (1.04, 1.11) 34.83
Adenocarcinoma
ICR 1.03 (0.87, 1.22) 1.47
MDACC 1.17 (0.96, 1.42) 1.12
IARC 1.08 (0.90, 1.29) 1.31
NCI 1.16 (1.06, 1.28) 4.13
Toronto 0.89 (0.58, 1.36) 0.25
GLC 0.90 (0.65, 1.25) 0.41
Harvard 0.89 (0.75, 1.07) 1.34
deCODE 1.08 (0.92, 1.28) 1.54
OncoArray 1.03 (0.98, 1.10) 8.40
Subtotal (I-squared = 26.5%, p = 0.209) 1.05 (1.00, 1.12) 19.97
Squamous cell carcinoma
ICR 1.08 (0.93, 1.25) 1.89
MDACC 1.14 (0.88, 1.46) 0.68
IARC 1.02 (0.89, 1.18) 2.06
NCI 1.12 (1.00, 1.25) 3.11
Toronto 1.14 (0.65, 2.03) 0.14
GLC 1.16 (0.77, 1.76) 0.26
Harvard 1.35 (1.04, 1.74) 0.66
deCODE 0.91 (0.71, 1.15) 0.75
OncoArray 1.06 (0.99, 1.14) 6.43
Subtotal (I-squared = 0.0%, p = 0.599) 1.08 (1.03, 1.13) 15.98
Ever smoking
IARC 1.12 (1.01, 1.25) 3.37
Toronto 1.12 (0.79, 1.58) 0.37
GLC 1.09 (0.80, 1.49) 0.46
Harvard 0.99 (0.83, 1.17) 1.43
MDACC 1.18 (1.00, 1.40) 1.49
ATBC 1.03 (0.88, 1.20) 1.73
CPSII 0.93 (0.74, 1.18) 0.80
EAGLE 1.27 (1.13, 1.43) 2.84
PLCO 1.23 (1.06, 1.43) 1.84
OncoArray 1.09 (1.04, 1.15) 9.89
Subtotal (I-squared = 29.1%, p = 0.177) 1.12 (1.06, 1.18) 24.21
Never smoking
IARC 1.09 (0.79, 1.49) 0.44
Toronto 1.04 (0.66, 1.64) 0.22
GLC 1.18 (0.58, 2.39) 0.09
Harvard 0.86 (0.55, 1.35) 0.22
CPSII 1.23 (0.77, 1.97) 0.20
EAGLE 0.95 (0.68, 1.33) 0.39
PLCO 1.08 (0.69, 1.68) 0.23
OncoArray 0.96 (0.86, 1.07) 3.23
Subtotal (I-squared = 0.0%, p = 0.939) 0.98 (0.90, 1.08) 5.02
.5
1
1.5
Odds ratio
rs198459

Supplementary Figure 2. Correlation between *GGA2* rs2285521 and the mRNA expression of *GGA2.* **a-f** Boxplot elements: *P* value was calculated from linear regression. Center-line indicates the median expression level across all participants in that group, and the hinges represent the lower (Q1) and upper (Q3) quartile, with lower whisker indicating the smallest value within 1.5 interquartile range (IQR) below Q1 and upper whisker indicating the largest value within 1.5 IQR above Q3 (**a-f**). *EARS2* rs6497670 in LC tissues from TCGA project (*GGA2* rs2285521 T>C exists complete LD with *EARS2* rs6497670 C>T): in additive and dominant models in lung cancer adjacent tissues (*P* = 0.438 and 0.573, respectively) (**a, b**); in additive and dominant models in NSCLC (LUAD and LUSC) tissues (*P* = 0.0128 and 0.00352, respectively) (**c, d**); in additive and dominant models in LUAD tissues (*P* = 0.22 and 0.0826, respectively) (**e, f**).

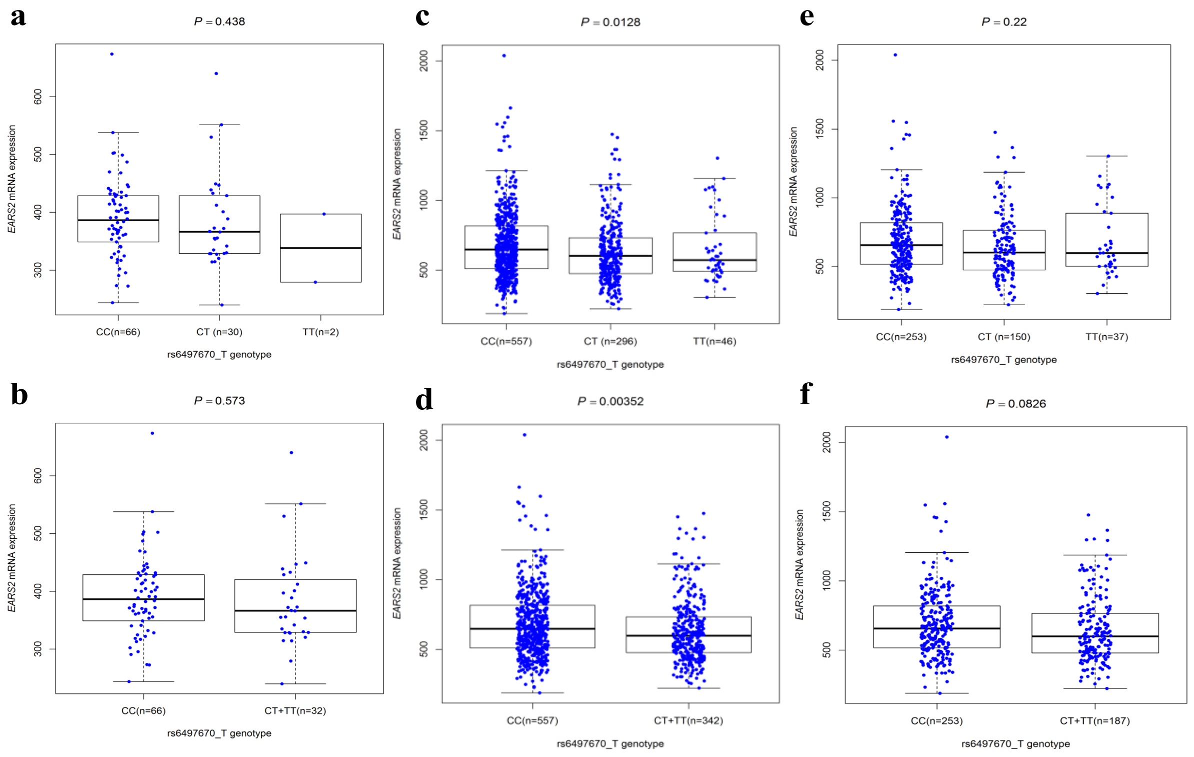

Supplementary Figure 3. Different mRNA expression analysis using data from the cancer microarray database (Oncomine: https://www.oncomine.org) showed the expression level of *JADE2, MYRF,* and *GGA2* in both LUAD and LUSC compared with normal tissues. **a, b** *JADE2* in LUAD (*P* = 0.039) (**a**) and in LUSC (*P* = 0.003) (**b**); **c, d** *GGA2* in LUAD (*P* = 2.83E-15) (**c**) and in LUSC (*P* = 5.66 E-12) (**d**); **e, f** *MYRF* in LUAD (*P* = 4.43E-10) (**e**) and in LUSC (*P* = 1.29E-26) (**f**). **a-f** Boxplot elements: *P* value was calculated from linear regression. Center-line indicates the median value across all participants in that group, and the hinges represent the lower (Q1) and upper (Q3) quartile, with lower whisker indicating the smallest value within 1.5 interquartile range (IQR) below Q1 and upper whisker indicating the largest value within 1.5 IQR above Q3.

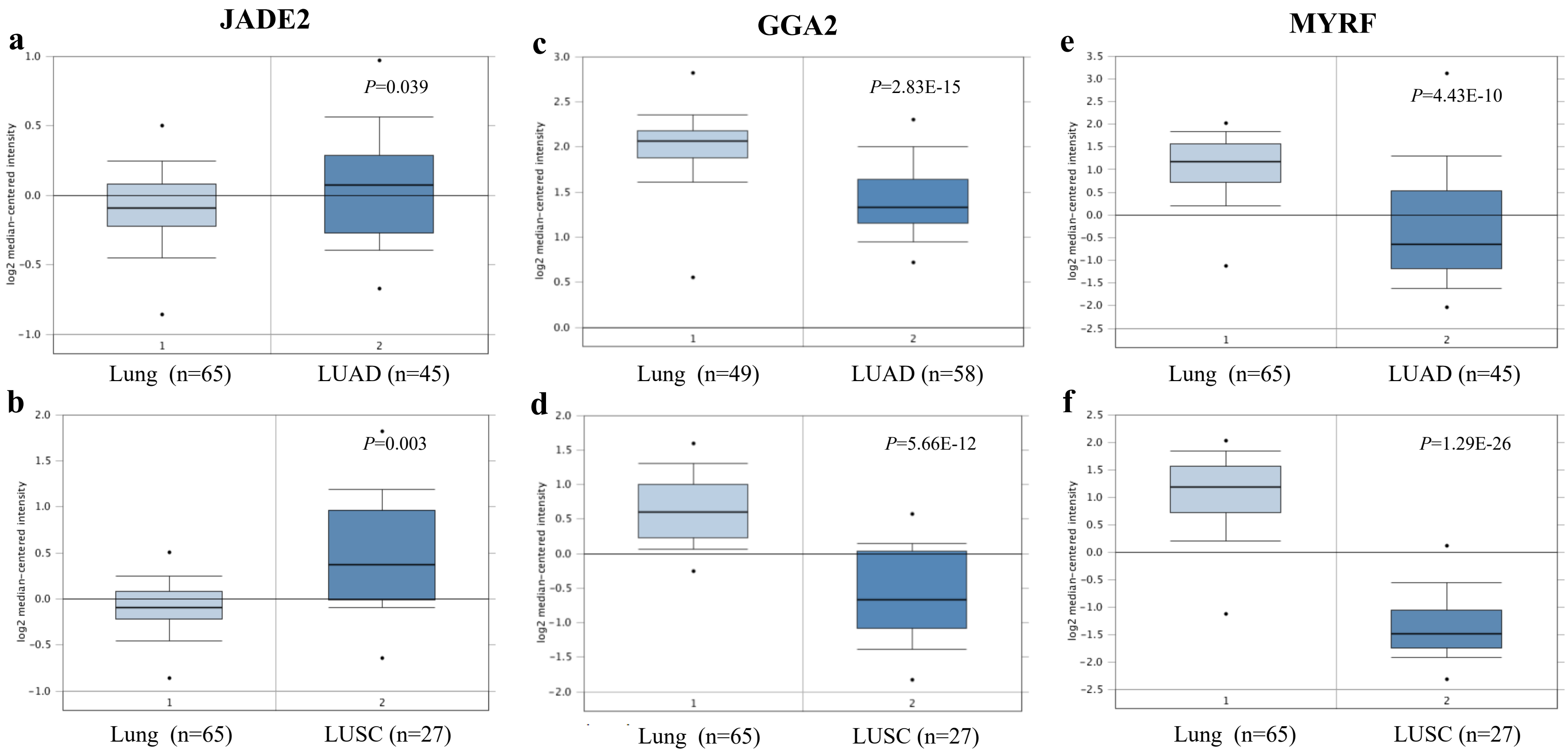

Supplement: Supplementary file 1 — Supplementary data [file 41698_2022_281_MOESM1_ESM.pdf]
